# The Population Structure of *Vibrio cholerae* from the Chandigarh Region of Northern India

**DOI:** 10.1371/journal.pntd.0002981

**Published:** 2014-07-24

**Authors:** Moataz Abd El Ghany, Jagadish Chander, Ankur Mutreja, Mamoon Rashid, Grant A. Hill-Cawthorne, Shahjahan Ali, Raeece Naeem, Nicholas R. Thomson, Gordon Dougan, Arnab Pain

**Affiliations:** 1 Pathogen Genomics Laboratory, Computational Bioscience Research Center, King Abdullah University of Science and Technology (KAUST), Thuwal, Kingdom of Saudi Arabia; 2 Department of Microbiology, Government Medical College Hospital (GMCH), Chandigarh, India; 3 The Wellcome Trust Sanger Institute (WTSI), the Wellcome Trust Genome Campus, Hinxton, Cambridge, United Kingdom; 4 Marie Bashir Institute for Infectious Diseases and Biosecurity and School of Public Health, University of Sydney, Sydney, Australia; 5 Bioscience Core Laboratory, King Abdullah University of Science and Technology (KAUST), Thuwal, Kingdom of Saudi Arabia; Oxford University Clinical Research Unit, Viet Nam

## Abstract

**Background:**

Cholera infection continues to be a threat to global public health. The current cholera pandemic associated with *Vibrio cholerae* El Tor has now been ongoing for over half a century.

**Methodology/Principal Findings:**

Thirty-eight *V. cholerae* El Tor isolates associated with a cholera outbreak in 2009 from the Chandigarh region of India were characterised by a combination of microbiology, molecular typing and whole-genome sequencing. The genomic analysis indicated that two clones of *V. cholera* circulated in the region and caused disease during this time. These clones fell into two distinct sub-clades that map independently onto wave 3 of the phylogenetic tree of seventh pandemic *V. cholerae* El Tor. Sequence analyses of the cholera toxin gene, the *Vibrio* seventh Pandemic Island II (VSPII) and SXT element correlated with this phylogenetic position of the two clades on the El Tor tree. The clade 2 isolates, characterized by a drug-resistant profile and the expression of a distinct cholera toxin, are closely related to the recent *V. cholerae* isolated elsewhere, including Haiti, but fell on a distinct branch of the tree, showing they were independent outbreaks. Multi-Locus Sequence Typing (MLST) distinguishes two sequence types among the 38 isolates, that did not correspond to the clades defined by whole-genome sequencing. Multi-Locus Variable-length tandem-nucleotide repeat Analysis (MLVA) identified 16 distinct clusters.

**Conclusions/Significance:**

The use of whole-genome sequencing enabled the identification of two clones of *V. cholerae* that circulated during the 2009 Chandigarh outbreak. These clones harboured a similar structure of ICE*Vch*Hai1 but differed mainly in the structure of CTX phage and VSPII. The limited capacity of MLST and MLVA to discriminate between the clones that circulated in the 2009 Chandigarh outbreak highlights the value of whole-genome sequencing as a route to the identification of further genetic markers to subtype *V. cholerae* isolates.

## Author Summary

*Vibrio cholerae* is a diarrheal pathogen that is responsible for substantial morbidity and mortality worldwide. Historically, seven pandemics of cholera have been recognized, with classical biotype strains associated with the sixth and the El Tor biotype with the seventh (current) pandemic. Recently multi-drug resistant El Tor variants expressing classical cholera toxin have replaced the original El Tor strains in many epidemics, and are sometimes associated with more severe diarrhea leading to a higher mortality rate. In regions that experience recurrent cholera outbreaks, such as Northern India, it is important to understand the nature of the circulating strains and establish how they are related to the strains circulating globally. Here, we have demonstrated that whole- genome sequencing is a valuable method to characterize *V. cholerae* isolates that circulated during the 2009 outbreak in the Northern Indian city of Chandigarh. Through comparative genomic analysis, we identified two clones that circulated during a single outbreak. Importantly, these clones contain significant differences in the structure of the cholera toxin gene and the *Vibrio* seventh pandemic island II. Our findings demonstrate the limitations of current molecular typing techniques and the importance of surveillance with whole-genome sequencing for identifying *V. cholerae* clades with distinct genomic signatures.

## Introduction

Cholera, caused by the Gram-negative bacterium *Vibrio cholerae*, is classically associated with rapidly dehydrating watery diarrhoea. Although more than 200 serogroups of *V. cholerae* have been identified; only the O1 and O139 serogroups are associated with epidemic and pandemic outbreaks. Historically, seven pandemics of cholera have been recognised with so called ‘classical’ strains associated with the first six and ‘El Tor’ stains with the seventh pandemic. El Tor isolates associated with clinical cholera fall into a monophyletic clade of highly related bacteria that have spread across the world in waves of outbreaks, recently reaching Haiti [1]. The main factor responsible for cholera disease is the cholera enterotoxin (CT). Variants of El Tor that produce classical type CT have been described [2] and such variants have been associated in some instances with more severe disease and higher case fatality rates [3].

Approximately one million cases of cholera were reported to the World Health Organization between 2009–11, associated with thousands of deaths [4], [5]. Moreover, the incidence of cholera has increased steadily in some regions of Africa and Asia over the past decade [4], [5]. These numbers are alarming because many cholera infections are not rapidly detected due to inadequate laboratory and epidemiological surveillance systems and economic, social and political issues associated with case reporting [5]. Cholera continues to be an important public health problem in India and during the period from 1997 to 2006, thousands of cases were reported annually [6]. A number of these Indian outbreaks have been associated with new El Tor variants [6].

Chandigarh, a city in northern India, has reported a number of outbreaks of cholera in recent years, associated with the monsoon season from June to October [7]–[11]. Located near the foothills of the Shivalik range of the Himalayas in northwest India, the Chandigarh health region occupies a 114-km^2^ footprint and is populated by over 900,000 people. We conducted a pilot study in Chandigarh using both molecular and whole- genome sequencing approaches for subtyping 38 *V. cholerae* isolates. Our aim was to understand the nature and diversity of the *V. cholerae* isolates circulating during the study period and determine how these isolates from the 2009 Chandigarh outbreak related to other current El Tor *V. cholerae* circulating elsewhere in the world. In particular, we wished to compare the resolution and accuracy afforded by some of the molecular approaches routinely used in subtyping of *V. cholerae* and the newer methods of whole-genome sequencing.

## Methods

### Ethics statement

The strain collection of *V. cholerae* described in the study was originally isolated by the Government Medical College Hospital (GMCH) for routine diagnostic purposes and was not experimental in nature. All the patient data used in this study were anonymised and unlinked.

### Characterization of bacterial isolates

A total of 38 isolates of *V cholerae* were obtained from patients with suspected cholera admitted to the GMCH, Chandigarh, India during a seasonal outbreak in 2009. Isolation and characterization of the bacteria were carried out according to recommended standard laboratory methods [12]. Bacterial isolates were screened by standard biochemical tests for the identification of *V. cholerae* [12], [13]. Serological identification was carried out by slide agglutination using commercially available antisera against *V. cholerae* O1 (Ogawa and Inaba) and O139 serogroups. Phage typing was performed based on previously described schemes [14], [15]. Susceptibility to antimicrobial agents was determined by the disk diffusion method and interpreted as recommended by Clinical and Laboratory Standards Institute (CLSI) with commercial antimicrobial disks, streptomycin (10 µg), tetracycline (30 µg), chloramphenicol (30 µg), sulfamethoxazole (25 µg), ciprofloxacin (5 µg) and trimethoprim (5.2 µg).

### Multi-Locus Sequence Typing (MLST) and Multi-Locus Variable-length tandem-nucleotide repeat Analysis (MLVA)

MLST was performed as previously described using nine genes: *dnaE*, *lap*, *recA*, *pgm*, *gyrB*, *cat*, *chi*, *rstR*, and *gmd* [16]. MLVA was performed as previously described [17] for five loci VC014, VC0436-37, VC1650, VC0171 and VCA0283. Briefly, each locus was amplified by PCR and the resulting fragments were purified and sequenced on an ABI3730 DNA analyser. MLVA alleles were distinguished by the number of tandem repeats as determined by Tandem Repeats Finder [18].

### Whole-genome sequencing

Genomic libraries were prepared for each sample, followed by multiplex sequencing on an Illumina GAIIx analyser at KAUST genomics core facility. The 75-base paired-end reads obtained were mapped against the reference *V. cholerae* N16961 El Tor (AE003852 and AE003853) using SMALT (http://www.sanger.ac.uk/resources/software/smalt) to obtain whole genome alignments for all the isolates used in this study [1]. Single nucleotide polymorphisms (SNPs) present in the regions that mapped to the N16961 genome were identified and these were filtered to ensure that they had a quality score more than 30 and were present in at least 75% of the mapped reads. These SNPs were then passed through a recombination removal filter as previously described [19] to exclude any high-density SNP clusters, which may be due to recombination. SNP information was used to estimate the maximum likelihood phylogeny using the default settings of RAxML v0.7.4 [20]. The numbers of SNPs on each branch were calculated by reconstructing all the polymorphic events on the tree using PAML [21]. The genome sequence of M66, a pre-seventh pandemic strain isolated from Indonesia in 1937 (accession numbers CP001233 and CP001234), was used as an out-group to root the phylogenetic tree [1]. Figtree (http://tree.bio.ed.ac.uk/software/figtree/), a phylogenetic tree reading program was used to visualise the tree and order the nodes. All the sequencing data has been submitted to the European Nucleotide Archive (sample accession number ERS353748 to ERS353785).

### Comparative genomics

Raw paired-end Illumina reads were assembled *de novo* using Velvet v0.7.03 [22] to generate a multi-contig draft genome for each of the 38 *V. cholerae* strains. The parameters were optimised to give the best kmer size and at least 20× coverage for each kmer. The high similarity between the seventh pandemic *V. cholera* strains allowed us to order contigs using ABACAS [23] in which the N16961 El Tor strain (AE003852 and AE003853) was used as a reference, followed by annotation transfer to each ordered draft genome. Finally, for each sample a genome comparison file was generated against N16961 using TBLASTX [24] and was viewed in Artemis Comparison Tool (ACT) for manual comparison of the genomes [25].

## Results

### Characterization of *V. cholerae* isolates from Chandigarh

**Figure 1:**
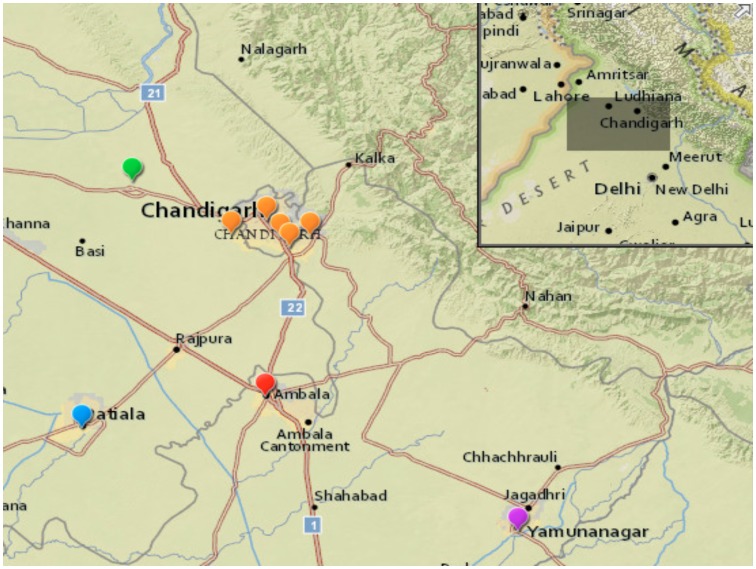
A Map of the Chandigarh region of India. The locations of the clinics where the *V. cholerae* isolates were obtained are illustrated. Each district has been assigned a different colour (Chandigarh; orange, Yamuna Nagar; violet, Ambala; red, Patiala; blue and Morinda; green). Source of the map is ESRI ArcGIS geomapping software.

In 2009, sixty-one culture -confirmed cases of cholera were reported from suspected cases admitted between June and October to the GMCH in Chandigarh (Figure 1). A total of 38 *V. cholerae* preserved as viable isolates were characterized first by microbiological, serological and molecular analysis, then by using whole-genome sequencing. All of the isolates were identified as *V. cholerae* O1 El Tor Ogawa and had originated from people inhabiting slum dwellings or suburban areas in and around Chandigarh (Figure 1, Table 1). Isolates were obtained from men, women and children aged between 1–38 years, with a median age of 8.6 years and the ratio of males to females was 1.6∶1. This age distribution of cholera cases reflects that observed across many outbreaks occurring in Chandigarh between January 1999 and December 2007 [11]. The isolates fell predominantly into the single phage types T2 [14] or T27 [15] depending upon the scheme used for typing. The antibiotic sensitivity profiles of the isolates from Chandigarh showed that all isolates tested, except isolate 5202 that was only resistant to trimethoprim, were multi-drug resistant. These resistant isolates displayed resistance to streptomycin, sulfisoxazole, sulfamethoxazole and trimethoprim but remained susceptible to tetracycline, chloramphenicol and ciprofloxacin (Table 1). A similar resistance pattern has been reported for *V. cholerae* El Tor causing outbreaks in many countries including Nepal, Cameroon, South Africa, Oman and India [26].

**Table 1:** Characterization of *V. cholerae* isolates from Chandigarh.

ID	Location	Clade	MLST type	MLVA type	Phage type	Antibiotic profile	Accession number
**4966**	Hallomajra	2	1	9-3-6-15-19	T2/27	A	ERS353748
**5046**	Mohali	2	1	9-3-6-15-19	T2/27	A	ERS353749
**5064**	NK	2	1	9-3-6-15-19	ND	A	ERS353750
**5157**	Hallomajra	2	1	9-3-6-15-19	T2/27	A	ERS353751
**5159**	Derabassi	2	1	9-3-6-15-19	T2/27	A	ERS353752
**5185**	Chandigarh	2	1	9-3-6-15-19	T2/27	A	ERS353753
**5186**	Hallomajra	2	1	9-3-6-15-19	T2/27	A	ERS353754
**5188**	Derabassi	2	1	9-3-6-15-19	T2/27	A	ERS353755
**5189**	Chandigarh	2	1	9-3-6-15-19	T2/27	A	ERS353756
**5197**	Derabassi	2	1	9-3-6-15-19	T2/27	A	ERS353757
**5198**	Derabassi	2	1	9-3-6-15-19	T2/27	A	ERS353758
**5202**	Derabassi	2	1	9-3-6-15-19	T2/27	B	ERS353759
**5235**	Derabassi	2	1	9-3-6-15-19	ND	A	ERS353760
**5238**	Chandigarh	2	1	9-3-6-15-19	T2/27	A	ERS353761
**5286**	Chandigarh	1	1	9-3-6-15-19	T2/27	A	ERS353762
**5365**	Derabassi	2	1	9-3-6-15-19	UT	A	ERS353763
**5366**	Hallomajra	2	1	9-3-6-18-19	T2/27	A	ERS353764
**5417**	Derabassi	2	1	9-3-6-15-18	T2/27	A	ERS353765
**5663**	Panchkula	2	1	9-3-6-15-19	ND	A	ERS353766
**6016**	Morinda	2	1	9-3-6-15-19	ND	A	ERS353767
**6099**	Chandigarh	2	2	10-5-7-11-19	ND	A	ERS353768
**6109**	Hallomajra	2	1	9-3-5-19-18	ND	A	ERS353769
**6111**	Chandigarh	2	1	9-3-5-20-18	ND	A	ERS353770
**6235**	Chandigarh	2	1	9-3-6-14-20	ND	A	ERS353771
**6236**	Hallomajra	2	1	9-3-5-19-19	ND	A	ERS353772
**6259**	Mohali	2	2	10-5-7-11-19	ND	A	ERS353773
**6361**	Mohali	2	1	10-3-6-15-19	ND	A	ERS353774
**6394**	Hallomajra	2	1	9-3-6-15-19	ND	A	ERS353775
**6554**	Hallomajra	2	1	9-3-5-19-18	ND	A	ERS353776
**6557**	Hallomajra	2	1	9-3-5-19-18	ND	A	ERS353777
**6702**	Chandigarh	2	1	9-3-6-15-20	ND	A	ERS353778
**6734**	Chandigarh	2	1	9-3-6-14-21	T2/26	A	ERS353779
**6801**	Panchkula	2	1	9-3-6-15-20	T2/21	A	ERS353780
**7683**	Ambala	2	1	11-7-7-14-21	T2/23	A	ERS353781
**7772**	Yammuna Nagar	1	1	11-3-6-19-18	ND	A	ERS353782
**7934**	NK	2	1	11-7-7-14-20	ND	A	ERS353783
**7994**	Patiala	1	1	11-7-7-14-19	ND	A	ERS353784
**9088**	NK	2	1	11-3-6-18-18	ND	A	ERS353785

A: resistance to Streptomycin, Sulfisoxazole/Sulfamethoxazole and Trimethoprim and susceptible to Tetracycline, Chloramphenicol, and Ciprofloxacin. B: Susceptible to all antibiotics used in the study except Trimethoprim. NK = not known, ND = not done.

### Phylogenetic analysis and relatedness of *V. cholerae* isolates from Chandigarh

**Figure 2:**
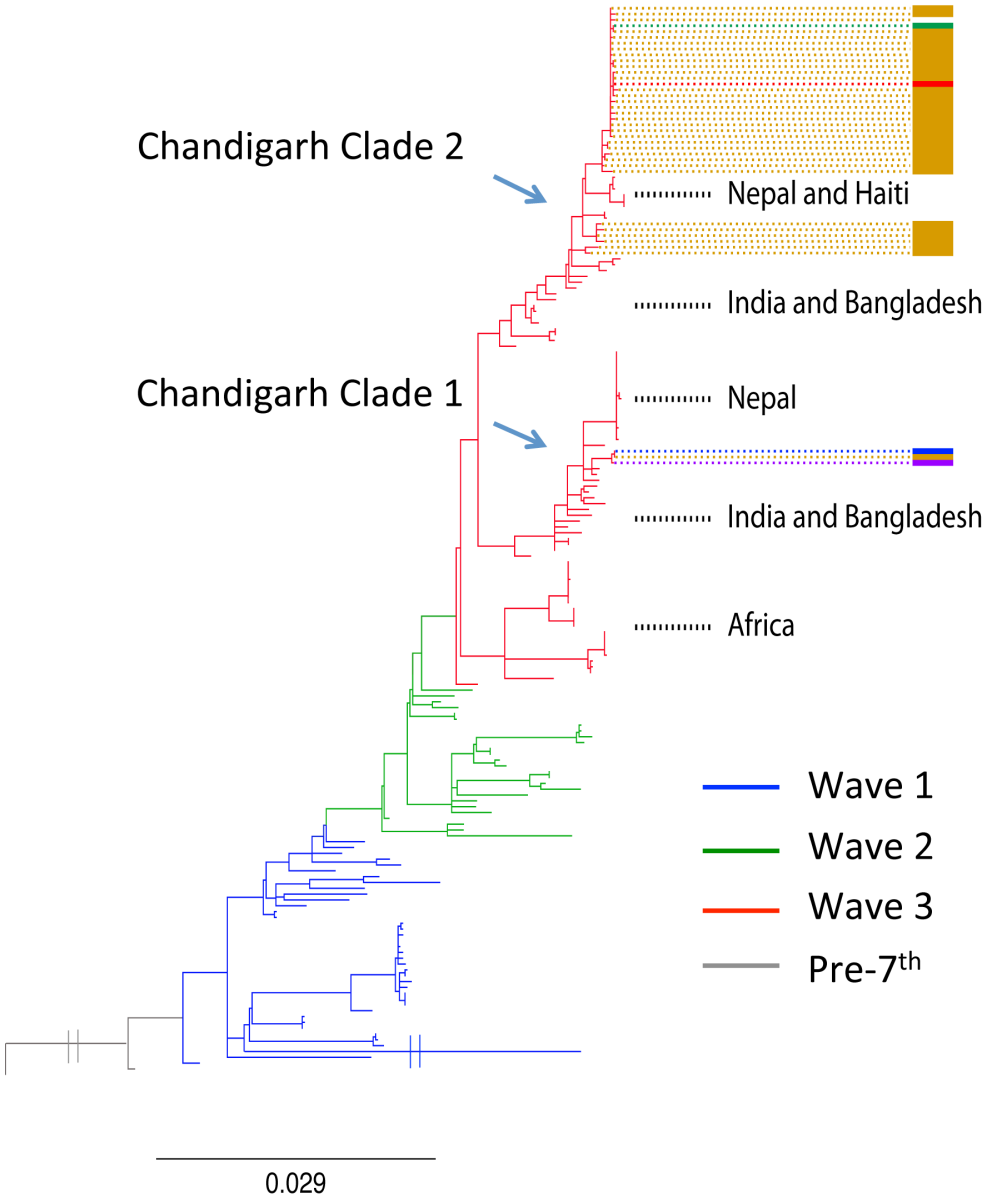
A SNP based maximum likelihood phylogeny of the seventh pandemic *V. cholerae* lineage. The phylogenetic tree shows the relation between the 2009 isolates from Chandigarh and the global collection of *V. cholera* genomes published by Mutreja et al [1]. The positions of the isolates from Chandigarh are indicated with colour bars on the right representing the district they were isolated from (see Figure 1). Other sub-clades that map close by on the tree to the Chandigarh isolates have been marked to highlight their global phylo-geographical context. The pre-seventh pandemic isolate M66 (2) was used as an out-group to root the tree. The scale given represents substitutions per variable site.

**Figure 3:**
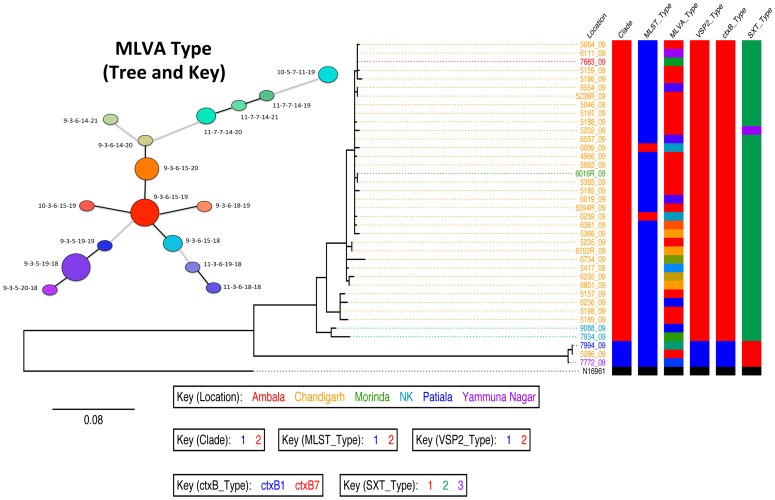
A SNP based maximum likelihood phylogeny of the Chandigarh *V. cholera*. The *V. cholerae* El Tor reference N16961 was used as the root of the tree. The colour key corresponds to location, clade, MLST type, VSP-II variants, *ctxB* alleles, SXT variants and the MLVA type. The scale of the tree is given as substitutions per variable site.

Whole-genome sequences for the 38 *V. cholerae* isolates from Chandigarh were used as a basis on which to establish a local bacterial population framework and to unequivocally place these isolates into the phylogenetic context of *V. cholerae* El Tor isolated across the world [1]. To achieve this, genomic DNA was initially isolated from the 38 *V. cholerae* isolates available to the study and these were sequenced using multiplex Illumina sequencing. SNPs present within these genomes were identified by mapping their sequence data against the genome of the *V. cholerae* El Tor reference N16961 (AE003852 and AE003853). In order to build a reliable phylogenetic tree, SNPs located within repetitive DNA or regions of likely recent recombination were removed and the final alignment based on 1776 SNPs was subjected to maximum likelihood phylogenetic analysis. The phylogenetic tree from this analysis is shown in Figure 2. All the Chandigarh *V. cholerae* isolates mapped onto the El Tor phylogenetic tree and clustered within wave-3 of the seventh pandemic. The Chandigarh isolates formed two distinct clades; named here Chandigarh clade 1 and 2, and these clades fell in two deeply separated sub-clades of wave-3 in the global seventh pandemic framework (Figure 2 and 3). Chandigarh clade 2 comprised the majority of the isolates except isolates 5286, 7772 and 7994, that formed clade 1. Chandigarh clade 1 isolates clustered with *V. cholerae* from India, Bangladesh and Nepal and isolates of clade 2 clustered on a distinct branch with recent *V. cholerae* outbreak isolates, including those from Nepal [27] and Haiti isolated in 2010 [28], [29].

### Structure variations in VSP-II, CTX and SXT confirm the presence of two *V. cholerae* El Tor sub-clades in Chandigarh

To examine the differences between clade 1 and clade 2 strains at a genomic level, assemblies of all the isolates were constructed and manual comparisons were made against the completed reference N16961 genome (AE003852 and AE003853). All the *V. cholerae* from Chandigarh possessed Vibrio pathogenicity island −1 (VPI-1), VSPII which are key genetic markers within the *V. cholerae* El Tor lineage [30], [31] and members of the R391 family ICE/SXT multiple antibiotic resistance cassettes. Chandigarh clade 2 isolates harboured a VSP-II variant 2 that was first described in the seventh pandemic *V. cholerae* O1 El Tor strain CIRS101. VSP-II variant 2 is characterized by a deletion in the loci VC0496 to VC0512 and this variant genetic structure is dominant in the most recent seven pandemic *V. cholerae* O1 El Tor isolates from many endemic sites [29], [31]. In contrast, Chandigarh clade 1 isolates harboured a VSPII variant 1 that has a similar structure to N16961 with a deletion in the loci VC0496, VC0497 and VC0498 (Figure 3).

Isolates from both of the clades found in Chandigarh harboured cholera toxin phages that are highly related to phages in other wave-3 *V. cholerae* of the seventh pandemic. These phage profiles were characterized by the presence of the El Tor *rstR* and classical *ctxB* alleles organized in a RS1–RS2 core array on the large chromosome. A similar CTX phage structure (*CTX-3b*) has also been reported as being present in ‘altered’ El Tor *V. cholerae* from Haiti [29], Nepal [27] and India [32]. However whole-genome analysis indicated that *V. cholerae* isolates from Chandigarh also formed two groups based on a *ctxB* polymorphism. In both clade-1 and 2 isolates, cholera toxin phages harboured mutations at base positions 20, 39 and 68 of the *ctx*B gene sequence. These mutations introduced asparagine, histidine and threonine respectively in clade-2 (the most recent *ctxB7* allele) and histidine, histidine and threonine respectively into clade-1 (*ctxB1* allele) toxin B subunit (Figure 3).

**Figure 4:**
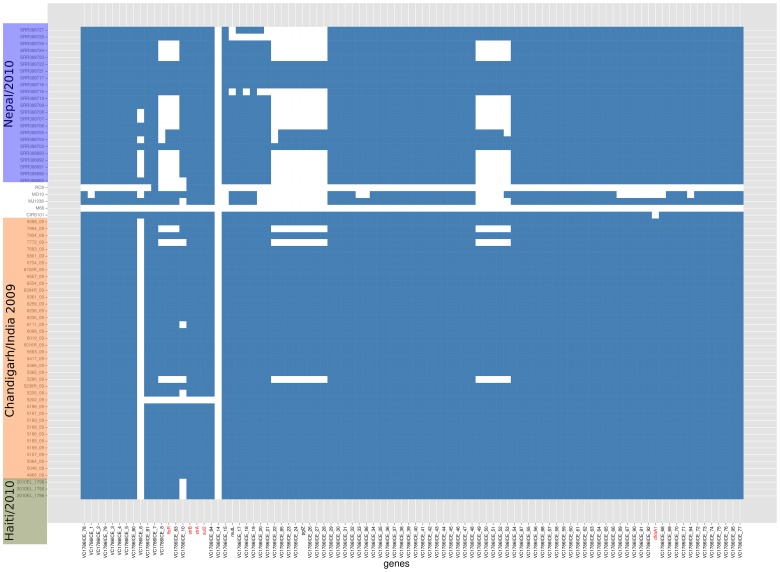
Structural variations in SXT among *V. cholerae* isolates. The structures of the SXT of *V. cholerae* isolates from Chandigarh were compared to published isolates from Nepal and Haiti. Reads were mapped to ICE*Vch*Hai1 (accession number JN648379). Plot showing presence (blue) or absence (white) of the genes of ICEVchHai1. The antibiotic resistance genes within the SXT cluster are highlighted in red.

Multi-drug resistance in *V. cholerae* is associated with the acquisition of mobile integrated conjugative elements (ICE) or SXT elements [33]. These elements have the ability to integrate and replicate within the host genome and can excise and transfer between bacteria by conjugation [34]. The acquisition of SXT by *V. cholerae* was first reported in *V. cholerae* O139 isolates from Madras, India [35]. The SXT element from one of these isolate, MO10, has been used as the reference for comparison with other SXT-like elements [36]. Recently, whole-genome sequencing of isolates from Haiti has identified a variant of SXT with minimal SNP differences compared to ICE*Vch*Ind5, (predominant in India) [37] and which was named ICE*Vch*Hai1 [38]. ICE*Vch*Hai1 harboured the antimicrobial resistance genes *strAB*, *sul2*, *dfrA1* and *floR* associated with resistance to streptomycin, sulfisoxazole/sulfamethoxazole, trimethoprim and chloramphenicol respectively [38]. All of the Chandigarh isolates sequenced in this study harboured these resistance genes except for isolate 5202 that had deletions of *straAB*, *sul2* and *floR*. Indeed, the Chandigarh SXT elements had only a few SNP differences compared to ICE*Vch*Hai1 (JN648379). At a sub-clade level, Chandigarh clade 2 isolates, (other than isolate 5202) shared highly related SXT structures with common deletions in loci VC1786ICE_6 and VC1786ICE_14. The SXT element of Chandigarh clade 1 isolates had a structural organization similar to the ICE*Vch*Hai1 but harboured significant deletions in loci VC1786ICE_9 (*floR*), VC1786ICE_14, VC1786ICE_22 to VC1786ICE_28, and VC1786ICE_49 to VC1786ICE_54. The variations in the structure of SXT among *V. cholerae* isolates from Chandigarh and those from recent isolates from Asia and South America are illustrated in Figure 4.

Taken together, the comparative genomic analysis strongly supports the whole genome based phylogenetic structure indicating that the 2009 outbreak in Chandigarh was in fact attributable to two independent lineages of *V. cholera*, both of which are traceable to positions within the wave 3 of the global seventh pandemic clone of *V. cholerae*.

### MLST and MLVA genotypes of *V. cholerae* in Chandigarh

Two molecular methods, MLST and MLVA, were used to initially genotype the *V. cholerae* isolates. MLST and MLVA are routinely used as tools to characterize *V. cholerae* isolates from global and local outbreaks and to investigate the origin of epidemics. MLST analysis of *V. cholerae* isolates for nine loci; *dnaE, lap, recA, pgm, gyrB, cat, chi, rstA and gmd* revealed little diversity, and clustered the isolates into two groups, a major cluster that contained the strains with sequence type 1 (ST1), and two isolates (6099, 6259) that fell into ST2 (Figure 3, Table 1). Two alleles were evident in five of the genes (*cat, chi, dnaE, lap, and recA*) and one gene *rstR* appeared to be significantly different in the minor cluster. Only three genes (*gmd*, *gyrB* and *pgm*) were conserved in all isolates.

MLVA is frequently used to discriminate between closely related *V. cholerae* isolates [39], [40]. Although there are several reports that conclude this technique has limited phylogenetic value [41] we looked for congruence between the whole-genome data and that of MLVA in an attempt to rationalise the data from both approaches. We performed MLVA profiling on the sequenced isolates within our collection by determining the number of repeats in five loci, VC0147, VC036-7, VC1650 VCA0171 and VCA0283. Using this approach we were able to identify 16 MLVA types (Figure 3 and Table 1). Three alleles were identified for each of the three loci on the large chromosome (VC0147, VC036-7 and VC1650) and six and four alleles were identified for the two loci on the small chromosome, VCA0171 and VCA0283 respectively. MLVA was able to discriminate between isolates within both cluster 1 and 2, although this variation did not completely correlate with whole-genome phylogeny (Figure 3 and Table 1). This was expected as evolution within these repeat regions is very rapid and is driven by different mechanisms from those associated with the accumulation of SNPs.

## Discussion

The seventh cholera pandemic associated with *V. cholerae* El Tor has now been ongoing for over half a century with continuing global impact. Recent analysis based on whole- genome sequencing has shown that the pandemic is associated with a monophyletic or highly clonal clade of *V. cholerae* O1 biotype El Tor that emerged in SE Asia and then spread in several waves around the world [1]. There are a number of examples of how whole-genome sequence technology can be used to study cholera within specific regions [27], [29], [42], [43]. Chandigarh, a province located in Northern India, continues to have a seasonal problem with endemic cholera. To understand basic information about the makeup and diversity of strains causing disease in this region we have analysed isolates of *V. cholerae* collected in outbreaks during a single cholera season in this region in 2009. A few important conclusions about the outbreaks are clear from this analysis.

Whole-genome sequencing clearly demonstrates that there were two distinct clades of *V. cholerae* circulating and causing disease in the region during 2009. Interestingly, looking at the map in Figure 1 there is some evidence that the two clades are covering different geographical areas within the study region, although further work would be required to validate this preliminary observation.

Although we cannot infer how long these clades have persisted in this region, both of the Chandigarh clades link directly into the global *V. cholerae* seven pandemic tree. Furthermore, Chandigarh clade 2 isolates fell higher up the pandemic seventh phylogenetic tree and were, more closely related to other *V. cholerae* isolates that have been circulated recently outside of the region such as the *V. cholerae* circulating in both Nepal and Haiti. Together this suggests that there have been at least two introductions of *V. cholerae* into this region and that the clade 2 isolates were the most recently introduced.

Our data provide a baseline to study the genomic changes that take place in the circulating *V. cholerae* clones in this region and link these changes with disease epidemiology and clinical features. It also demonstrates the importance of conducting further high-throughput sequencing studies, as the clades will evolve in future. In addition, the study shows the limited capacity of MLST and MLVA to establish epidemiological links between isolates. MLST distinguished two sequence types among the 38 isolates that did not correspond to the clades defined by whole-genome sequencing. In contrast for the same isolates, MLVA showed sixteen distinct genotypes. When compared to the MLVA data it was interesting that MLVA had significant, if somewhat lower resolution than whole genome-sequencing. However, although it did facilitate isolate discrimination, it could not provide a true phylogenetic picture of the outbreak. This, limits MLVA's utility to resolve outbreaks even when isolates are collected over a single cholera season.

In conclusion, using whole-genome sequencing linked to phenotypic analysis we were able to detect two clones of *V. cholerae* that circulated during the 2009 outbreak in Chandigarh. These clones mainly differed in the structure of CTX and VSPII. This study reemphasizes the value of whole-genome sequencing over other molecular approaches for investigating outbreaks of *Vibrio cholerae*.
